# Human oral microbiome and its influence on mental health and brain disorders

**DOI:** 10.3934/microbiol.2025013

**Published:** 2025-04-14

**Authors:** Alejandro Borrego-Ruiz, Juan J. Borrego

**Affiliations:** 1 Departamento de Psicología Social y de las Organizaciones, Universidad Nacional de Educación a Distancia (UNED), Madrid, Spain; 2 Departamento de Microbiología, Universidad de Málaga, Málaga, Spain

**Keywords:** human oral microbiome, mental health, oral diseases, oral–gut–brain axis, brain disorders

## Abstract

The human oral microbiome can affect brain functions directly through the trigeminal nerve and olfactory system and indirectly via the oral–gut–brain axis. However, the potential link between the oral microbiome and mental health remains an area that requires further investigation. Taking into consideration that gut microbiota dysbiosis plays a role in the onset and progression of several mental disorders, as well as the potential influence of the oral microbiome on mental health via direct pathways, the present narrative review explores the role of the human oral microbiome in health and disease, along with the factors that affect its composition, with a particular focus on its potential impact on mental health, including its involvement in a range of mental disorders and brain-related conditions, such as Alzheimer's disease, Parkinson's disease, autism spectrum disorder, anxiety, depression, stress, bipolar disorder, Down's syndrome, cerebral palsy, epilepsy, and schizophrenia. Chronic oral diseases can impair the oral mucosal barrier, allowing microorganisms and endotoxins to enter the bloodstream, triggering systemic inflammation, and affecting the blood–brain barrier. This pathway can lead to neuroinflammation and cognitive dysfunction and contribute to adverse mental health effects. Additionally, translocation of oral bacteria to the gut can drive persistent inflammation and thereby affect brain health. Multiple studies suggest a potential relationship between the oral microbiome and several mental disorders, but further research is needed to strengthen the evidence surrounding these associations and to fully clarify the underlying mechanisms linking the oral microbiome to these conditions. Given the promising implications, future research should focus on elucidating the biological mechanisms through which alterations in the oral microbiome influence the development and progression of determinate neurodegenerative and neuropsychiatric disorders. Additionally, identifying reliable biomarkers linked to the oral microbiome could enhance early detection, diagnosis, and monitoring of these conditions.

## 1. Introduction

The human mouth presents a range of substrates, including cheeks, gums, teeth, and tongue, which possess differences in chemistry, stability, and topography, providing distinct habitats for microbial communities [1]. A broad variety of microorganisms that colonize the oral cavity engage in complex interactions among themselves and with their host, typically resulting in a balanced coexistence, which is known as a state of eubiosis. Nevertheless, diverse factors such as dietary patterns, inadequate oral hygiene, tobacco use, and the effect of specific medications can disturb this fragile homeostatic balance, which is known as dysbiosis, potentially contributing to the onset of oral diseases and increasing the risk of systemic conditions, including mental health disorders [2]–[5].

Host-related factors can favorably influence the oral microbiome (OM), fostering diversity and equilibrium among distinct microbial species, which leads to a state of symbiosis and lack of disease [6]. Moreover, the human OM impacts a wide range of pivotal host functions, mediating a range of immunological, metabolic, and physiological processes, such as the maturation of the adaptive and innate immune system in the host [7].

Current research indicates that co-evolution has led to the functional integration of the host and OM, a relationship that underpins the concepts of holobiont or superorganism, highlighting that we function as a unified biological entity [8]. In this regard, the host gains advantages such as resistance to infection, achieved through the inhibition of colonization by pathogenic microorganisms [9], maturation of the adaptive and innate immune systems, and adjustment of immune response patterns to maintain a balance between anti-inflammatory and pro-inflammatory dynamics [10],[11]. Nevertheless, it is plausible that many of these advantages also extend to OM [12].

Following the achievements of the Human Microbiome Project [13] and the Human Oral Microbiome Database (eHOMD) [14], studies in oral microbiology have advanced to a new stage, with more than 2000 reference genomes of oral microorganisms currently compiled in the database [15]. This data has been leveraged to investigate the functions and varieties of microorganisms within the oral cavity, construct a chart of the oral microbiota distribution, and evaluate the composition of each microorganism [16]. However, despite these advancements, the potential link between OM and mental health remains an area that requires further investigation. For this reason, the present narrative review explores the role of the human OM in health and disease, along with the factors that affect its composition, with a particular focus on its potential impact on mental health, including its involvement in a range of mental disorders and brain-related conditions, such as Alzheimer's disease, Parkinson's disease, autism spectrum disorder, anxiety, depression, stress, bipolar disorder, Down's syndrome, cerebral palsy, epilepsy, and schizophrenia.

## 2. The oral microbiome in health

The oral environment is a diverse and conducive ecosystem for the proliferation of several microbial groups due to its adequate temperature, humidity, and nutrients provided by the gingival crevicular fluid and salivary proteins [6],[17]. OM consists of approximately 1000 bacterial species, although viruses, fungi, protozoa, and archaea are also present in smaller proportions [18]. The major bacterial phyla in the oral cavity include members of Actinomycetota, Bacteroidota, Fusobacteriota, Bacillota, Pseudomonadota, and Spirochaetota [15]. Interestingly, there are also other minority phyla and candidate phyla such as Chloroflexota, Chlorobiota, candidate phylum Gracilibacteria (GN02/BD1-5), Synergistota, candidate phylum SR1, candidate phylum radiation Saccharibacteria (TM7), and candidate phylum WPS-2 [19]–[22].

Although oral archaea are regarded as non-pathogenic, some of them have been described in several oral diseases, including periodontitis and caries [23],[24], belonging to the species *Methanobrevibacter oralis, M. cuticularis, M. filiformi, M. ruminantium*, and *M. arboriphilius* [25]. The oral mycobiome includes the genera *Aspergillus*, *Aureobasidium*, *Candida*, *Cladosporium*, *Cryptococcus*, *Fusarium*, *Gibberella*, *Malassezia, Penicillium*, *Rhodotorula*, *Saccharomyces*, and *Schizophyllum*, which have been primarily isolated from the oral rinses, supragingival plaque, hard palate, and saliva [26],[27]. The oral virome is composed of bacteriophages and eukaryotic viruses [28]. Most of the eukaryotic viruses found in the oral cavity are linked to various diseases, such as papilloma, condyloma, and focal epithelial hyperplasia caused by the human papillomavirus (HPV) infection [29],[30]; herpes genitalis, herpes labialis, mucocutaneous orofacial disease, herpetic gingivostomatitis, and herpetic paterecleris caused by herpes simplex virus [31],[32]; and HIV-related lesions that include Kaposi sarcoma, non-Hodgkin lymphoma, and necrotizing ulcerative periodontitis [33],[34]. The other viral components of OM are bacteriophages, whose main role is bacterial lysis [35]–[37]. It has also been proposed that the viruses in saliva serve as carriers for pathogenic genes within the oral environment [38]. Compared to other microbial groups, protozoa are the minority component of OM. In general, oral protozoa are non-pathogenic commensals typically found in individuals with exiguous oral hygiene or complete edentulism [34]. However, *Entamoeba gingivalis* and *Trichomonas tenax* have been reported to be implicated in several periodontal conditions (e.g., periodontitis and gingivitis) [39].

Members of the Bacteria domain comprise the greatest microbial diversity and abundance of OM. Studies originating from the eHOMD have shown that oral bacteria are located at specific sites within the oral cavity, including the cheek, gingival sulcus, teeth, tongue, soft and hard palate, and tonsils, as well as planktonic bacteria present in salivary fluid [2],[4],[40],[41]. Furthermore, the supragingival, subgingival, interdental, and tongue regions are different oral areas that possess a distinctive microbiota composition [42]–[45]. Within the mouth, the predominant microbial habitats are governed by the genus *Streptococcus*, followed in abundance by *Haemophilus* in the buccal mucosa, *Actinomyces* in the supragingival plaque, and *Prevotella* in the subgingival plaque. In saliva, *Streptococcus, Prevotella, Veillonella, Neisseria*, and *Haemophilus* are the most abundant genera [46].

**Figure 1.**
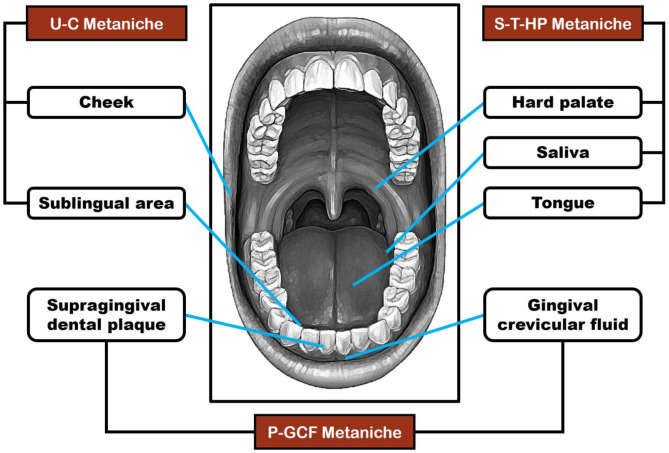
Metaniches of OM associated with anatomically oral regions (according to [2]).

According to Cho et al. [2], the oral cavity can be organized into three metaniches. The P-GCF metaniche is formed by the bacterial genera on supragingival dental plaque and gingival crevicular fluid, dominated by *Actinomyces* and *Corynebacterium* (phylum Actinomycetota), *Capnocytophaga* and *Tannerella* (phylum Bacteroidota), *Fusobacterium* and *Leptotrichia* (phylum Fusobacteriota), and *Aggregatibacter* (phylum Pseudomonadota). The S-T-HP metaniche is formed by the bacteria present in the saliva, tongue, and hard palate, which is dominated by the genera *Granulicatella* and *Veillonella* (phylum Bacillota), *Alloprevotella, Porphyromonas* and *Prevotella* (phylum Bacteroidota), and *Neisseria* (phylum Pseudomonadota). Finally, the U-C metaniche, which includes the cheek and sublingual area, is mainly dominated by *Gemella* and *Streptococcus* (phylum Bacillota), and *Actinobacillus* and *Haemophilus* (phylum Pseudomonadota). Figure 1 shows the metaniches of OM linked to specific oral anatomical regions.

On the other hand, Li et al. [47] proposed that OM are distributed into three niches: saliva, tooth surface, and soft tissue surface, with the former including the buccal mucosa, gingiva, tongue, and palate [48]. Members of Bacillota are more abundant in the saliva, while the members of the phyla Actinomycetota and Fusobacteriota are more prevalent in dental plaque [49]. A study of the salivary microbiota in a Qatari population found that the most abundant genera in the salivary microbiota were *Gemella*, *Haemophilus, Neisseria, Porphyromonas, Prevotella, Streptococcus*, and *Veillonella* [50]. Nevertheless, the microbial composition of saliva from Japanese subjects consisted of *Granulicatella adiacens, Neisseria flavescens*, *Rothia mucilaginosa*, *Streptococcus mitis*, and *Streptococcus salivarius* [51]. These variations can be affected by dietary, environmental, and genetic factors [52]. Furthermore, saliva exerts a great influence on the microbial adhesion and colonization processes [53] and encompasses a range of defense proteins that contribute to oral mucosal immunity, including α-amylase, antimicrobial peptides, lysozyme, mucin, statherin, peroxidases, and salivary immunoglobulins [54].

The tooth surface provides an ideal environment for bacterial growth and plaque formation. Dental plaque has been shown to harbor higher α-diversity, microbial richness, and uniformity than tongue and saliva samples [55]. Anaerobic bacteria such as *Actinomyces, Fusobacterium*, and *Veillonella* are primarily present in the subgingival plaque [28]. The microbial composition on the tooth surface is also shaped by the physiology and anatomy of the perigingival region and the tooth surfaces. Moreover, the crown surface of a single tooth can be categorized into distinct regions with notable variations in bacterial composition [56],[57]. For example, *Streptococcus* spp. are found on the labial surface of canines and incisors, but rarely on the lingual surface [54],[57]. Although *Streptococcus* is the predominant genus in mucosal tissues [28], there are differences between the components of oral mucosal tissues. The tongue exhibits a higher diversity and greater density of microorganisms compared to other mucosal surfaces [48]. In this respect, facultative and obligate anaerobes, such as *Actinomyces, Haemophilus, Leptotrichia, Neisseria, Porphyromonas, Prevotella, Streptococcus*, and *Veillonella*, are the major microorganisms on the tongue [55].

The interplay among microbial communities of different species can be mutually beneficial, as the presence of one microorganism creates an environment conducive to another, and the metabolic byproducts of one species serve as nutrients for another, demonstrating syntrophism [18]. As a result, these microbial groups engage in complex metabolic exchanges, making it essential to adopt a metabolomic perspective when studying OM [58]. In contrast, antagonistic interactions are also established in OM, facilitated by adhesion-binding sites and direct competition for nutrients, by the secretion of antibiotic substances and bacteriocins, and by the induction of conditions such as low pH that are unfavorable for other microbiota members [12]. Simultaneous horizontal gene transfer and homologous recombination contribute to the diversity and interdependence of members of OM [59].

In reaction to factors like chewing, salivary flow, and mastication, these sites within the mouth display distinct physicochemical characteristics, such as oxygen tension, nutrient content, mechanical forces, and pH levels, all of which influence the selection of microorganisms for each specific oral niche. In addition, structures such as the teeth and tongue offer a physical foundation that supports the establishment of oral microbial biofilms.

### 2.1. Oral microbial biofilms

Oral microbial biofilms are intricate ecological systems that contain a variety of oral microorganisms and may be linked to several oral diseases [47]. The initial colonizing bacteria are often several *Streptococcus* species, including *S. gordonii, S. mitis, S. oralis*, and *S. sanguinis*, which specifically bind to their corresponding salivary receptors via surface adhesins [60]. This binding is pivotal for the development of multispecies biofilms and relies on the adhesins present on one bacterium's surface and the complementary polysaccharide-containing receptors found on the other species [61]. In its mature state, the biofilm consists of various microorganisms, proteins, lipids, extracellular polymeric substances, and extracellular polysaccharides from saliva and food [62],[63]. As the oral microbial biofilms mature, the predominant bacteria shift from *Streptococcus* in the initial stage to strict anaerobes such as *Capnocytophaga, Fusobacterium, Porphyromonas, Prevotella, Veillonella*, and *Actinomyces* in the later stages [64]–[67]. In particular, *Fusobacterium nucleatum* plays a crucial role in establishing interdependent interactions with other microbiota members, functioning as a link between early and late colonizers [68]. This is largely due to the strong and extensive adhesive properties of its outer membrane proteins [69], which mediate colonization with Gram-positive bacteria such as *Staphylococcus aureus, S. gordonii*, and *Streptococcus mutans*, and even with the yeast *Candida albicans* [47],[70],[71]. The close physical proximity between microorganisms in the biofilm allows the existence of communication between either the same or different bacterial species, facilitated by chemical signals that are synthesized and released by the bacteria in the biofilm. These signals can be categorized into two types: one associated with cell density and quorum sensing, and the other involving signals generated by bacteria at different stages of their growth [72],[73].

## 3. Factors inducing alterations in the oral microbiome

Several factors may influence the composition of OM, such as age, diet, drugs, exposure to toxic substances, use of antimicrobials, genetic predisposition, and pregnancy [74]. Food components, including sugars, fats, and vitamins, are considered to be the most influential factors that affect microbial composition, including both the community structure and diversity of OM [47]. Some studies have reported that OM of individuals in lower socioeconomic conditions tends to have reduced biodiversity, with higher concentrations of bacteria such as *Achromobacter xylosoxidans*, *Aggregatibacter segnis*, and *Neisseria* cluster II. Conversely, higher socioeconomic status individuals exhibit elevated levels of *Prevotella histicola*, *Megasphaera micronuciformis*, *Veillonella atypica*, *Veillonella parvula, Rothia mucilaginosa, F. nucleatum, G. adiacens*, and *Tannerella forsythia* [75],[76]. Table 1 shows several factors promoting changes in OM.

**Table 1.** Factors promoting changes in the oral microbiome (OM).

Factors	Major findings	Reference
Health conditions	Poor: -Lower microbial diversity -Increases abundance of *Aggregatibacter segnis*, *Achromobacter xylosoxidans*, and *Neisseria* cluster II Good: -Increases abundance of *Megasphaera micronuciformis*, *Veillonella atypica*, *V. parvula, Rothia mucilaginosa, Prevotella histicola, Fusobacterium nucleatum, Granulicatella adiacens*, and *Tannerella forsythia*	[75],[76]
Antibiotic treatment	-Decreases Actinomycetota members	[76],[77]
	-Reduces diversity index -Decreases abundance of Actinomycetota, Bacteroidota, and Fusobacteriota members -Increases abundance of members of Pseudomonadota and *Aggregatibacter aphrophilus*	
	-Decreases the abundance of *Neisseria, Streptococcus*, and *Veillonella* and of members of *Rikenellaceae*	[78]–[80]
	-Increases antibiotic-resistant genes	[81]
Dietary patterns	-Higher proportion of *Streptococcus* in breastfed infants -Increases abundance of *Actinomyces* and *Prevotella* in formula-fed infants	[82]
	-Positive correlation between carbonated beverages and the abundance of Bacteroidota, Pseudomonadota, *Fusobacterium*, and *Veillonella*	[83]
	-Alcohol consumption increases *Streptococcus mutans* and inhibits *Fusobacterium* activity	[84]
Smoking	-Decreases abundance of *Streptococcus sanguinis* and *S. parasanguinis* -Increases abundance of *Fusobacterium naviforme* and *F. nucleatum*	[85]
Smoking	-Decreases abundance of Pseudomonadota, *Neisseria subflava*, *Corynebacterium, Capnocytophaga, Peptostreptococcus*, and *Leptotrichia* -Increases abundance of Bacillota, Actinomycetota, *Atopobium* and *Streptococcus*	[86]
	-Increases microbial diversity -Increases abundance of Fusobacteriota, Actinomycetota, *Streptococcus*, *Prevotella*, and *Veillonella*	[87]
Pregnancy	-Increases abundance of *Neisseria, Porphyromonas*, and *Treponema* -Decreases abundance of *Streptococcus* and *Veillonella*	[88]
	-Increases abundance of *Porphyromonas gingivalis* and *Aggregatibacter actinomycetemcomitans*	[89]

### 3.1. Antibiotic treatment

Antibiotics such as amoxicillin, azithromycin, ciprofloxacin, and clindamycin have the most significant effects on the microbiota, resulting in a reduction of Actinomycetota [76]. These antibiotics impact both the functions and composition of OM while also inducing particular metabolic alterations during antibiotic treatments [79],[90]. A prospective cohort study conducted in patients with endodontic infection treated with amoxicillin found that antibiotic therapy decreased the microbial diversity index, with the relative abundance of members of Actinomycetota, Bacteroidota, and Fusobacteriota decreasing after 1 week of treatment [77]. In contrast, the abundance of members of Pseudomonadota and *Aggregatibacter aphrophilus* was detected at 1 month after antibiotic therapy. Other studies have also shown that amoxicillin reduces the abundance of the genera *Neisseria, Streptococcus*, and *Veillonella* and members of the family *Rikenellaceae* in OM [78]–[80]. In addition to these antibiotic effects on OM, the increase in the abundance of genes linked to antibiotic resistance in the buccal cavity is also important [81]. It has been observed that certain prevalent oral fungi show resistance to antifungal azoles [91], with genes related to resistance against fluconazole and ketoconazole being significantly expressed in *C. albicans* obtained from individuals suffering from periodontal disease [92].

### 3.2. Dietary patterns

The OM of infants who were breastfed showed a greater proportion of *Streptococcus*, while infants who were formula-fed exhibited a greater abundance of *Actinomyces* and *Prevotella*. In addition, breastfed and mixed-fed infants were less likely to develop oral candidiasis than solid food-fed infants [82]. This significant difference in the oral microorganisms between breastfed and formula-fed infants may be related to the production of hydrogen peroxide, an antimicrobial substance [93]. In adults, dietary shifts can also alter the OM and help prevent the growth of microbial pathogens [94]. Several nutrient components, such as dietary fiber, various types of fatty acids, and carbohydrates are associated with OM diversity and community structure [47],[95]. For example, the intake of carbonated beverages is positively associated with the abundance of members of the phyla Bacteroidota and Pseudomonadota and the genera *Fusobacterium* and *Veillonella* and with the occurrence of caries [83]. Sugar alcohols, including sorbitol, erythritol, and xylitol, are commonly used as sugar substitutes for preventing caries, although they also alter the composition of the salivary microbiota [96]. Similar to sugar, overconsumption of alcohol, with the exception of red wine, can disrupt the balance of OM by increasing the levels of Gram-positive bacteria such as *S. mutans*, while simultaneously inhibiting the *Fusobacterium* activity due to elevated acetaldehyde concentrations [74],[84],[97].

### 3.3. Smoking

Cigarette smoking introduces its own bacterial load to the mouth, including species such as *Bacillus* spp. and *Clostridium* spp. [86]. The toxic substances in cigarettes can lead to the depletion of beneficial oral bacteria, facilitate pathogen colonization, and ultimately result in disease, either directly or through mechanisms like biofilm formation and immunosuppression [85],[98],[99]. These changes increase processes such as salivary acidification and oxygen depletion, leading to the development of anaerobic bacteria [76]. Among smokers, a higher presence of detrimental commensals such as *S. sanguinis* and *Streptococcus parasanguinis*, alongside abundant anaerobic microorganisms like *F. nucleatum* and *Fusobacterium naviforme*, was reported. In turn, this was accompanied by a greater taxonomic diversity and richness, which closely reflected a microbiota composition typically linked to disease, even in clinically healthy individuals [85]. In particular, periodontal pathogens belonging to members of phylum Synergistota and the genera *Cardiobacterium, Selenomonas*, and *Fusobacterium*, along with respiratory pathogens that belong to the genera *Haemophilus* and *Pseudomonas*, colonized the initial biofilms of smoking individuals [100].

Other studies on smoking subjects have reported that the abundance of Pseudomonadota, *Neisseria subflava*, and *Corynebacterium* in OM are substantially decreased, while members of Bacillota and Actinomycetota are increased. Furthermore, the *Leptotrichia*, *Peptostreptococcus*, and *Capnocytophaga* genera showed reduced abundance in current smokers compared to those who had never smoked, while the *Streptococcus* and *Atopobium* genera were found to be more prevalent [86]. In a systematic review that included 36 studies, Senaratne et al. [87] concluded that tobacco users exhibited increased bacterial richness and diversity, with substantial enrichment of Fusobacteriota and Actinomycetota at the phylum level, and *Veillonella*, *Prevotella*, and *Streptococcus* at the genus level.

Interestingly, one study revealed that microbial richness and diversity showed no significant variations when comparing smokers to non-smokers [101]. This discrepancy was explained to be due to several factors, including diet, ethnicity, and environment. Research has demonstrated that following a specific duration of smoking cessation, OM composition in former smokers aligns closely with that of never-smokers, suggesting that quitting smoking aids in reestablishing a balanced microbiome [86],[98],[102]. Recent evidence has shown that the potential impacts of smoking on OM can encompass impairing immune defenses, forming anaerobic conditions, altering salivary pH and bacterial adhesion, and introducing antimicrobial effects through harmful chemicals in cigarettes [86],[98].

### 3.4. Pregnancy

In the course of pregnancy, women experience significant and complex hormonal fluctuations that influence the composition of OM [103]. Several studies have found an elevated risk of periodontal disease during pregnancy, along with alteration in OM composition [104],[105]. In general, the abundance of *Treponema*, *Porphyromonas*, and *Neisseria* increases during pregnancy, while the abundance of *Veillonella* and *Streptococcus* decreases [88]. The presence of *Porphyromonas gingivalis* in periodontal pockets has been associated with microbial invasion of the amniotic cavity, which may contribute to preterm labor [106]. Moreover, an increase in *Aggregatibacter actinomycetemcomitans* and *P. gingivalis* during pregnancy has been noted in the gingival sulcus compared to non-pregnant women [89]. In the last trimester, the most abundant bacterial genera are *Capnocytophaga sputigena, Lachnoanaerobaculum* (formerly *Eubacterium*) *saburreum, F. nucleatum, F. nucleatum* subsp. *polymorphum, Leptotrichia buccalis, Prevotella melaninogenica, Parvimonas micra, Prevotella intermedia, S. aureus, Streptococcus anginosus, Streptococcus intermedius, Streptococcus oralis, S. mutans, S. gordonii, Selenomonas noxia*, and *V. parvula* [74],[76],[103],[107],[108].

## 4. Oral diseases

It has been demonstrated that disease processes alter the microbial diversity of healthy OM [109]–[111]. One study reported that α-diversity was lowest in caries-associated microbiota compared to that of healthy OM, with statistically significant differences observed between caries and healthy OM saliva samples. Regarding β-diversity, the microbial composition of caries samples was distinct from that of saliva samples classified as caries-free or caries-active, particularly when bacterial abundance was taken into account [109]. In the case of periodontal disease, a comparative analysis of the oral mucosa and subgingival plaque from healthy individuals and periodontitis patients revealed increased microbial diversity in the periodontitis group [110]. Another study [111] evaluating α-diversity in saliva and subgingival samples found no significant differences between healthy and periodontitis groups. However, within the periodontitis group, higher diversity was observed in saliva and subgingival areas compared to supragingival sites. Moreover, β-diversity analysis further confirmed distinct microbial community clustering between healthy and diseased groups in both supragingival plaque and saliva samples, although no significant differences in overall diversity or evenness were detected.

### 4.1. Dental caries and tooth decay

Dental caries occur when acids generated by the breakdown of dietary carbohydrates by commensal oral bacteria cause demineralization of the tooth surface. Key contributors to the development of caries encompass microbial dental plaque, consumption of sugars, and an acidic oral environment [112]. Nevertheless, saliva helps counteract these effects by buffering acids, raising pH levels, and restoring lost ions through a process termed remineralization [113]. Without adequate removal of biofilm (due to poor oral hygiene), bacteria metabolize sugars, releasing acids that erode tooth enamel and promote caries formation [112].

Acid generation in dental biofilms is predominantly linked to lactobacilli and streptococci, with the latter dominating. Oral streptococci exhibit notable variability in their acidogenicity and tolerance to acidic conditions. *S. mutans* and *Streptococcus sobrinus* are capable of catabolizing sucrose by converting it primarily into lactate, as opposed to generating a blend of alcohols and weaker acids observed in other species [12]. Moreover, certain strains of *S. mitis* exhibit acid production comparable to streptococci of the mutans group at pH 5.5, whereas other strains of this species, along with *S. gordonii*, *S. sanguinis*, and *S. oralis* subsp. *dentisani*, are significantly less acidogenic [114]. Nevertheless, the pH within dental biofilms is influenced not only by acid generation but also by the accumulated metabolic activities of the microbial community. Determinate oral microorganisms such as *Streptococcus cristatus*, *S. gordonii*, *S. oralis* subsp. *dentisani*, *S. parasanguinis*, and *S. sanguinis* generate ammonia as a byproduct of arginine metabolism [115]. Additionally, *Actinomyces* and *Haemophilus parainfluenzae* contribute to ammonia production through urease activity [116], enhancing the buffering capacity of the biofilm's microenvironment. Consequently, elevated alkali generation driven by arginine deiminase and urease pathways within the mouth is strongly related to caries resistance [117].

An acidic condition in the oral environment triggers the production of enzymes and provokes changes in the microbial composition, favoring the selection of acid-tolerant aerobic microorganisms. Although *S. mutans* has been considered uniquely responsible for caries in research using culture methods, more recent studies have found that other species are also involved, including *Lactobacillus* spp., *Bifidobacterium* spp., and *Actinomyces* spp. [118]. The application of molecular techniques has opened new perspectives in understanding which bacteria are linked to distinct kinds and phases of dental caries, which can be divided into three main types: (i) early caries, characterized by the appearance of white spot lesions on the enamel, which are the initial stages of tooth decay; (ii) dentin caries, which occurs when the decay extends beyond the enamel and reaches the underlying dentin, leading to deeper tissue damage; and (iii) root caries, which specifically affect the cementum or dentin of the tooth roots, typically in individuals with gum recession or exposed roots. In contrast, in childhood caries, several studies have shown a differential involvement of OM. Ma et al. [119] reported various genera such as *Actinomyces*, *Porphyromonas*, and *Streptococcus*, which were linked to severe caries in early childhood, constituting potential biomarkers of dental caries in the initial dentition. Other studies have reported the occurrence of the genera *Dialister*, *Filifactor*, *Prevotella*, and *Lactobacillus* in the pathogenesis of dental caries in Chinese children [120]. In Canadian children with severe early-childhood caries, *Veillonella* spp., *Porphyromonas* spp., and *S. mutans* were more abundant, while *S. gordonii* and *S. sanguinis* dominated in the non-caries group [121].

Tooth decay refers to the irreversible loss of minerals from the tooth's hard tissues, including the enamel and dentin. It is primarily caused by the organic acids produced by bacteria within the dental plaque. These acids are generated through the anaerobic breakdown of dietary sugars, especially sucrose, which occurs after the intake of sugary foods [122].

### 4.2. Periodontal diseases

The primary driver of periodontal diseases is the accumulation of dental plaque on the tooth surfaces, often stemming from inadequate oral hygiene. These conditions are classified into two main types: gingivitis and periodontitis [121]. Periodontal diseases generally arise from microbial biofilm (dental plaque) and involve a range of bacterial species that proliferate in number due to plaque dysbiosis [123]. Persistent inflammation may result in the establishment of periodontal pockets, which disrupt the local nutrient balance and redox state, fostering greater microbial diversity and richness within the biofilm, ultimately exacerbating dysbiosis [124].

A healthy subgingival microbiome is predominantly composed of Gram-positive bacteria such as *Actinomyces* spp. and *Streptococcus* spp., which serve as initial colonizers and play a critical role in forming the foundational layer of dental plaque. *F. nucleatum*, the second most prevalent species, functions as an intermediary colonizer, facilitating connections between diverse bacterial species as the plaque matures [46]. In addition, Gram-negative bacteria like *Capnocytophaga* spp. and *Veillonella* spp. contribute significantly to biofilm formation. Furthermore, Gram-positive species such as *Rothia* spp. and Corynebacteria have been recognized for their role in spatial arrangements within biofilms [125]. The onset of gingivitis induces a shift in the subgingival microbiome, marked by an increase in Gram-negative bacteria such as *Selenomonas* spp., *F. nucleatum* subsp. *polymorphum*, and *Prevotella* spp., accompanied by a decline in Gram-positive species. This microbial transition correlates with elevated inflammatory cytokine levels in gingival crevicular fluid [126],[127].

Gingivitis arises from the continuous formation of microbial biofilms, a process that may be intensified by localized risk factors, including calculus accumulation, misaligned teeth, and iatrogenic restorations. These elements are considered primary contributors to the onset and progression of gingivitis [128]. Without proper intervention, gingivitis can advance to periodontitis, which is a severe condition mainly marked by the destruction of alveolar bone and by the development of periodontal pockets [129]. Key anaerobic bacterial species implicated in driving the inflammatory response that underpins gingivitis are Actinomycetota, *Campylobacter*, *Eiknella*, *Fusobacterium*, *Capnocytophaga*, and *Prevotella* [130].

As previously mentioned, periodontitis constitutes a chronic inflammatory disease of bacterial origin that results in irreversible damage of the periodontal connective tissues, resorption of alveolar bone, as well as tooth loss [129]. The most prevalent forms of periodontitis are linked to anaerobic, Gram-negative bacteria, including *P. gingivalis*, *P. intermedia*, and *Treponema denticola*, which produce virulence factors like cysteine protease (PrtH), dentilisin, and gingipain, which exhibit potent proteolytic activity [40],[131]. The immune system reacts strongly to the presence of these microorganisms in the gum, triggering the release of pro-inflammatory cytokines such as tumor necrosis factor-α (TNF-α) [132]. Periodontitis is further characterized by elevated serum levels of C-reactive protein (CRP) and by a reduction in anti-inflammatory markers like interleukin (IL)-10 [133]. Clinically, this manifests initially as severe inflammation of the gums, progressing to the irreversible destruction of the tooth-supporting structures, including the alveolar bone and periodontal ligament [134].

### 4.3. Oral candidiasis

Among healthy subjects, *Candida* species are naturally present on mucosal surfaces, including the oral cavity, gastrointestinal tract, nasal passages, reproductive organs, and skin. Oral candidiasis, often called “thrush”, is a fungal infection of the oral mucosa primarily provoked by the overgrowth of *Candida* spp., with *Candida albicans* being the most prevalent species. Factors that contribute to its development encompass the use of specific medications, impaired salivary gland function, diets high in carbohydrates, and dental prosthetics [135]. Saliva contains antimicrobial agents such as amylase, lactoferrin, lysozyme, glycosylated proline-rich proteins, and antibodies specifically targeting *Candida* [136]. In addition, dental materials and structures can create a conducive microenvironment for *Candida* growth, promoting colonization in conditions with low oxygen and acidic pH. This process is further facilitated by diets rich in carbohydrates that enhance *Candida* adherence to oral epithelial cells. Moreover, other risk factors linked to oral candidiasis include cancer, cigarette smoking, diabetes, and compromised immune function [74].

### 4.4. Oral lichen planus

Oral lichen planus (OLP) is a persistent inflammatory condition that primarily affects the oral mucosa and is classified as a mucocutaneous disease. Although its etiology and pathogenesis remain largely unclear, OLP has been associated with several underlying conditions and triggers, including hypersensitivity reactions to dental materials, autoimmune disorders, microbial infections (bacterial and viral), certain medications, and vaccines [137]. Research into OM's role in OLP is limited. However, Bornstein et al. [138] identified elevated bacterial counts of *C. sputigena, Eikenella corrodens, Lactobacillus crispatus, Mobiluncus curtisii, Neisseria mucosa, Prevotella bivia, P. intermedia, Streptococcus agalactiae*, and *Staphylococcus haemolyticus* at OLP lesion sites. Similarly, Ertugrul et al. [139] noted increased colonization by pathogens such as *P. gingivalis*, *A. actinomycetemcomitans*, *T. denticola*, *P. intermedia*, and *T. forsythia* in individuals with OLP compared to controls. Furthermore, Choi et al. [140] observed a reduction in *Streptococcus* alongside a proliferation of bacteria linked to gingivitis and periodontitis in OLP lesions. More recently, Yan et al. [141] found a notable rise in the relative abundance of *Pseudomonas, Aggregatibacter, Campylobacter*, and *Lautropia* in OLP.

## 5. Oral microbiome in systemic diseases

Although this topic is beyond the scope of this review, we summarize recent data from scientific literature linking the onset or exacerbation of specific systemic diseases to disruptions in OM. This microbiome, along with associated inflammatory mediators, can reach the systemic organs through two main pathways: the circulatory system and the digestive tract. The first route, bloodstream transmission, occurs due to the anatomical proximity of periodontal pockets to blood vessels. The second route involves OM reaching the digestive tract through alimentary dissemination [142]. However, the exact mechanisms of invasion need to be determined in each case [143]. Table 2 shows how OM is involved in several systemic diseases.

**Table 2.** Major oral microbiome (OM) involved in several systemic diseases.

Phylum	Genera	Systemic diseases	Reference
Bacillota	*Lactobacillus*	Cardiovascular	[144]
	*Selenomonas*	Systemic lupus erithematosus Lung cancer	[145],[146]
	*Solobacterium*	Bacteremia	[147]
	*Streptococcus*	Diabetes Cardiovascular Chronic obstructive pulmonary Colorectal cancer	[144],[148]–[150]
	*Veillonella*	Hirschsprung's disease-associated enterocolitis Cardiovascular Lung cancer Inflammatory bowel disease	[146],[151]–[153]
Bacteroidota	*Capnocytophaga*	Diabetes Lung cancer	[146],[154]
Bacteroidota	*Porphyromonas*	Diabetes Cardiovascular Rheumatoid arthritis Adverse pregnancy outcomes Inflammatory bowel disease Pancreatic cancer Esophageal cancer	[155]–[162]
	*Prevotella*	Metabolic dysfunction Cardiovascular Rheumatoid arthritis Inflammatory bowel disease Systemic lupus erithematosus	[151],[163]–[166]
	*Tannerella*	Diabetes Cardiovascular Rheumatoid arthritis Esophageal cancer	[160],[167]–[169]
Fusobacteriota	*Fusobacterium*	Diabetes Cardiovascular Chronic obstructive pulmonary Adverse pregnancy outcomes Ulcerative colitis Colorectal cancer	[170]–[175]
Pseudomonadota	*Aggregatibacter*	Diabetes Cardiovascular Inflammatory bowel disease Pancreatic cancer	[176]–[180]
	*Campylobacter*	Inflammatory bowel disease	[181]
	*Haemophilus*	Chronic obstructive pulmonary	[182]
	*Moraxella*	Chronic obstructive pulmonary	[183]
Spirochaetota	*Treponella*	Diabetes	[169]

## 6. Oral microbiome, mental health, and brain-related disorders

Although many studies establish a clear association between the gut microbiome and several mental disorders [184], evidence on the influence of OM and its pathophysiological mechanisms in relation to neuropsychiatric disorders is more limited [185]. However, several studies have identified specific OM in individuals with mental disorders, leading to the hypothesis of a relationship between these two components. This hypothesis assumes that the central nervous system (CNS) is able of altering the oral environment to favor the selection of specific microbial groups, as is the case for the gut microbiota [186], as well as influence poor oral condition in patients with mental disorders [187]. Several reviews have collected the involvement of OM or oral diseases in diverse neurodegenerative, neuropsychiatric, and psychological conditions such as Alzheimer's disease (AD), Parkinson's disease (PD), autism spectrum disorder (ASD), depression, anxiety, and psychological stress [188],[189]. In addition, other mental health disorders and adverse brain-related conditions have been occasionally implicated in OM dysbiosis, such as epilepsy and cerebral palsy, schizophrenia, and bipolar disorder [190].

**Figure 2.**
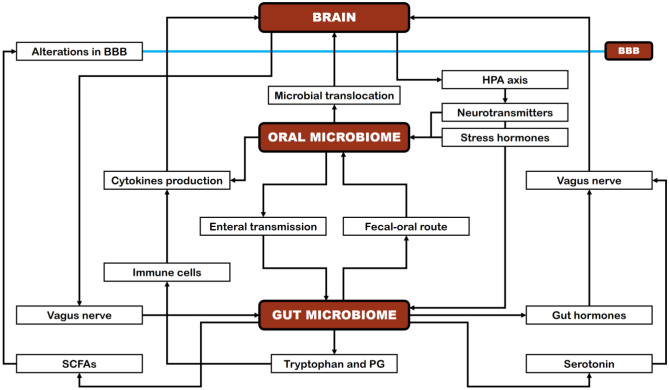
Schematic representation of the oral–gut–brain axis. BBB: blood–brain barrier; PG: peptidoglycans; SCFAs: short-chain fatty acids.

It has been postulated that OM may directly and indirectly affect brain function through several mechanisms. For instance, directly through microbial translocation to the brain via the trigeminal nerve and olfactory system, which connects the oral cavity to the olfactory bulb in the brain [191],[192]. Persistent oral diseases may impair the oral mucosal barrier [193], facilitating microorganism or lipopolysaccharide (LPS) access to bloodstream through mechanical damage (brushing or chewing), contributing to a severe inflammatory response by stimulating endothelial cells that express TNF-α and IL-1β receptors, which signal perivascular macrophages, driving neuroinflammation and activation of the hypothalamic–pituitary–adrenal (HPA) axis [194]. Moreover, these inflammatory mediators lead to the breakdown of the blood–brain barrier (BBB) [195], which is linked to inflammation and cognitive decline in certain mental conditions [196],[197]. *P. gingivalis* is recognized for its ability to invade the bloodstream and travel to the brain, where it establishes colonies and segregates neurotoxic proteases known as gingipains [198]. Gingipains are implicated in disrupting the processing of the transmembrane protein β-amyloid precursor protein (APP), which plays a pivotal role in maintaining neuronal growth and protection, as well as synaptic stability [199]. Additional pathways for entry into the brain include the choroid plexus, circumventricular organs, and leptomeninges [197],[200]. On the other hand, OM can indirectly influence the brain function via the oral–gut–brain axis [201]. The translocation of oral microbial species to the gut via saliva can affect the shape of the gut microbiota [202]. Therefore, the mouth could act as a reservoir for gut pathogenic microorganisms that can trigger the gut immune system and provoke persistent inflammation [203],[204], thereby leading to increased intestinal epithelial permeability [205],[206]. Systemic inflammation can produce changes in neurovascular function, resulting in increased BBB permeability, decreased nutrient delivery, and accumulation of toxins in the brain [198],[207]. Figure 2 shows a schematic representation of the oral–gut–brain axis.

The increased relative abundance of oral bacteria in fecal samples can be explained by two main hypotheses. The *expansion hypothesis* suggests that oral bacteria invade the gut ecosystem and proliferate, whereas the *marker hypothesis* proposes that these bacteria merely pass through the gut, with their relative increase indicating a depletion of native gut microbiota. In this regard, the detection of oral bacteria in feces associated with inflammation, intestinal diseases, and depletion of the gut microbiome supports the marker hypothesis [208]. Costa et al. [209] investigated the relationship between OM and the gut microbiome by sequencing the V4 region of the 16S ribosomal RNA gene and performing an amplicon sequence variant (ASV) analysis. Although the composition of both microbiomes was distinct, they identified a subset of 61 ASVs shared between the oral and gut microbiomes of the same individuals. Of these, 38 ASVs (62%), encompassing 23 genera, were more prevalent in oral samples, whereas 17 ASVs (28%), representing 12 genera, were more abundant in the gut. Only three genera, *Limosilalactobacillus* and *Weissella* (phylum Bacillota) and *Atopobium* (phylum Actinomycetota), showed similar abundance in both microbiomes. The predominance of shared ASVs in the oral cavity suggests that oral-to-gut transmission is the primary route of bacterial translocation, a process likely more common in healthy individuals. Despite the continuous ingestion of oral bacteria via saliva and food, most fail to reach the gut in a viable form due to physiological barriers such as gastric acidity, bile acids, the host immune response, and competition with resident gut bacteria. These factors make colonization of the gut by oral bacteria largely inefficient. However, Kageyama et al. [210] suggested that this translocation may be influenced by aging and the accumulation of dental plaque. Certain oral bacterial species have been identified in cases of intestinal disorders and proposed as potential biomarkers for gastrointestinal diseases [211]. These findings highlight the importance of the oral-gut microbiome axis and indicate that maintaining a balanced microbial environment in both ecosystems could be pivotal for gastrointestinal health. Interestingly, alterations in OM of patients with neurodegenerative disorders closely resemble changes observed in the gut microbiome of individuals with AD and PD [212]. Similarly, gut microbiome dysbiosis in neuropsychiatric conditions has been linked to shifts in bacterial genera within OM, particularly in ASD, depression, and anxiety, while distinct patterns were observed in schizophrenia [184].

### 6.1. Neurodegenerative disorders

#### 6.1.1. Alzheimer's disease (AD)

AD is a progressive neurodegenerative condition marked by cognitive decline, memory dysfunction, and impaired activities in daily living [213]. AD is primarily characterized by the accumulation of amyloid beta (Aβ) plaques and neurofibrillary tangles (NFTs) in the brain, which disrupt normal neural function and lead to the gradual deterioration of cognitive abilities. Amyloid products are part of the host's immune response to pathogens in both the peripheral systems and CNS. These products are generated through the protease-mediated cleavage of the APP. APP is a type I transmembrane protein that can be processed by several enzymes, such as γ-secretase, α-secretase, and β-secretase [214]. Epidemiological studies have suggested the existence of a relationship between periodontal disease together with OM and cognitive decline, dementia, and the development of AD [133],[215]–[217]. For example, a nationwide retrospective cohort study conducted in Taiwan revealed that subjects with persistent gingivitis and periodontitis were more likely to develop dementia than those who had healthy gums [218]. In addition, Chen et al. [219] noted that chronic periodontitis was related to a more elevated risk of developing AD.

A large body of evidence has established a direct causal link between various microbiota of OM and AD. *P. gingivalis* has been shown to induce the accumulation of Aβ plaques and NFTs following an experimental oral infection in mice, which also demonstrated that oral administration of gingipain inhibitors reduced *P. gingivalis* levels and decreased Aβ42 generation in mouse neural tissue [220]. Other studies have reported that the persistent systemic *P. gingivalis* infection induced Aβ accumulation in inflammatory monocytes/macrophages through the stimulation of CatB/NF-κB signaling [221], as well as in the human neuroblastoma cell line SH-SY5Y [222]. Moreover, the administration of *P. gingivalis* induced pro-inflammatory responses, Aβ production in the brain, and impaired cognitive performance in Sprague-Dawley rats and C57BL/6J and SAMP8 mice [223],[224]. Intraperitoneal injection of *P. gingivalis* LPS into male C57BL/6J and SAMP8 mice resulted in a decrease of the neprilysin expression in the hippocampus [223], and lower levels of neprilysin have been linked to increased Aβ accumulation in AD [225],[226].

*A*. *actinomycetemcomitans* has been involved in AD via its capacity to provoke inflammatory responses, influence the CNS, and induce the secretion of β-amyloid [227]. In addition, *A. actinomycetemcomitans* can enhance the production of inflammatory cytokines and toll-like receptors in brain cells, resulting in the elevated expression of pro-inflammatory cytokines like TNF-α, IL-6, and IL-1β [228]. *T. denticola*, together with *T. forsythia* and *P. gingivalis*, form the “red complex”, a well-established polybacterial pathogenic consortium in periodontitis. These bacteria play a crucial role in local dysbiosis in OM and lead to the disruption of the immune response of the host [229]. Importantly, *Treponema* species have been found in the brains of individuals with AD [230]. Animal studies have shown that oral inoculation of *T. denticola* in C57BL/6 mice results in the accumulation of Aβ1-42 within the hippocampus and in neuronal apoptosis, indicating a possible connection with memory dysfunction [231],[232]. *F. nucleatum* has been proposed as a potential microorganism involved in the development and pathogenesis of AD. In this regard, oral infection with *F. nucleatum* may provoke heightened learning impairment in AD-like rats. Furthermore, *F. nucleatum* exposure increased the Aβ1-42 and p-Tau181 expression in AD-like rats [233]. Nevertheless, *F. nucleatum* infection has been associated with increased local or systemic inflammation, which affects the permeability of the BBB. This subsequently triggers CNS and systemic immune activation, neuroinflammation in the CNS, the deposition of Aβ plaques within the brain, and the development of AD [234]. In addition, Watanabe et al. [235] reported that Cnm-positive *S. mutans* were more frequently found in the saliva of individuals with cerebral microbleeds. Moreover, cognitive function was lower in individuals with Cnm-positive *S. mutans* that expressed collagen-binding activity. Cerebral microbleeds are considered a risk factor for dementia, and the collagen-binding Cnm protein expressed by Cnm-positive *S. mutans* contributes to the formation of these cerebral microbleeds [236].

Additionally, research has established further links between AD and OM. For instance, Liu et al. [237] reported a lower abundance and biodiversity of the salivary microbiota in individuals with AD compared to those of healthy controls. These authors noted relatively greater levels of *Sphaerochaeta*, *Leptotrichia*, and *Moraxella*, and also a substantial reduction in the abundance of *Rothia* in the saliva of subjects with AD. In another study, patients with AD had lower microbial diversity than controls, with a significantly increased abundance of members of the order Lactobacillales and the family *Streptococcaceae*, and a significantly decreased abundance of the genus *Fusobacterium* [238]. In a cohort study conducted in Canada, Cirstea et al. [239] found that the OM of AD patients had higher microbial diversity, with a lower abundance of the *Streptococcaceae* and *Actinomycetaceae* families and a greater abundance of members of the family *Weeksellaceae*. The periodontitis-associated oral microorganism *P. gingivalis* was more prevalent in AD patients. On the other hand, Fu et al. [240] found increased frequencies of *Capnocytophaga* spp., *Eubacterium infirmum*, *Prevotella buccae*, and *Selenomonas artemidis* in the AD group. More recently, Issilbayeva et al. [241] conducted a case-control study of OM diversity in AD patients compared to healthy Central Asian seniors and reported that the OM of AD had a higher microbial diversity, with an increase in members of Bacillota and a decrease of Bacteroidota members in the AD group. At the genus level, these authors found five prevalent genera in AD patients: *Bacteroides* (phylum Bacteroidota), *Methylorubrum* (formerly *Methylobacterium*; phylum Pseudomonadota), and *Anaerostipes*, *Lactobacillus*, and *Shuttleworthia*, belonging to the phylum Bacillota. However, other authors found no significant differences in microbial diversity between AD and healthy subjects [242].

The exact mechanisms by which the oral pathogens influence the onset and progression of AD remain not fully elucidated. The accumulation of essential pathogenic elements, including tau and Aβ, in the brain may stem from diverse factors [234]: (i) the migration of microbial products from the mouth to the brain, resulting in continuous activation of CNS immune cells; (ii) oral inflammation impacting neuroinflammation in the CNS; (iii) entry of OM into the brain due to increased BBB permeability; (iv) the movement of oral pathogens or their byproducts (bacterial peptidoglycan and LPS) into the peripheral system, thereby triggering pro-inflammatory monocyte/macrophage migration into the brain; and (v) the initiation of AD pathology from a precise anatomical area, owing to the direct connection between the trigeminal nuclei, locus coeruleus, proprioceptors, and periodontal free nerve endings with the CNS.

#### 6.1.2. Parkinson's disease (PD)

PD is marked by the progressive loss of dopaminergic neurons within the substantia nigra. The condition manifests through both motor and non-motor symptoms, including loss of smell (anosmia), rapid eye movement sleep disturbances, gastrointestinal issues such as constipation, mood disorders like depression, autonomic dysfunction, and cognitive decline [243]. One study examined differences in OM composition and the extent of oral inflammation between individuals with PD and healthy controls [244]. No differences in the periodontal parameters were found, but the levels of IL-1β, IL-1 receptor antagonist, and TNF-α were significantly higher in PD patients compared to the healthy controls [244]. These authors stated that salivary and subgingival dental plaque microbiota also diverged between individuals with PD and controls, including the higher abundance of *Kingella oralis*, *S. mutans*, *Scardovia* spp., *Actinomyces AFQC_s*, *Veillonella AFUJ_s*, and members of taxa *Lactobacillaceae*, Negativicutes, and Bacillota, whereas *Lachnospiraceae AM420052_s*, *Treponema KE332528_s*, and candidate phylum Absconditabacteria (formerly phylum SR1) were less abundant.

In a cross-sectional case-control study employing 16S rRNA gene sequencing, Rozas et al. [245] identified the OM from soft and hard tissues of individuals with PD compared to those of healthy controls. The study revealed substantial distinctions in soft tissue β-diversity and a higher prevalence of potentially pathogenic oral species in individuals with PD, such as *Lactobacillus* spp., *T. forsythia*, and *P. intermedia*. Subjects with PD also presented heightened levels of endogenous opportunistic pathogens, including *Streptococcus constellatus*, *Streptococcus pneumoniae*, and *Mycoplasma orale*. In addition, the hard tissue bacterial communities of PD patients showed no overall community differences (β-diversity) compared to the controls, although a total of 12 OTUs were substantially more abundant in patients with PD, including the genera *Fusobacterium*, *Capnocytophaga*, *Alloprevotella*, *Prevotella*, and the species *Campylobacter rectus*. In another study, it was reported that ligature-induced periodontitis involving subgingival plaque (LIP-SP) was shown to exacerbate dopaminergic neuron loss, microglial activation, and motor dysfunction in mice with PD induced by 1-methyl-4-phenyl-1,2,3,6-tetrahydropyridine (MPTP) [246]. These authors demonstrated that the oral pathogens *S. mutans* and *V. parvula* require the presence of periodontitis to aggravate neurodegeneration and motor dysfunction in mice with PD induced by MPTP. This effect was linked to microglial activation and T helper 1 (Th1) cell infiltration in the cervical lymph nodes, brain, colon, and ileum of mice with PD. In addition, neutralizing IFNγ provided a protective effect on dopaminergic neurons in PD mice treated with *S. mutans* and *V. parvula*.

### 6.2. Neuropsychiatric and psychological conditions

#### 6.2.1. Autism spectrum disorder (ASD)

ASD is a multifaceted developmental neuropsychiatric condition marked by multiple levels of social interaction and communication difficulties. Children with ASD tend to exhibit low oral hygiene and a greater incidence of dental caries than controls [247]. This deterioration in oral hygiene may be due to some of the typical behavioral symptoms and manifestations of the condition among children, such as personal negligence, communication limitations, eating habits, self-injurious behavior, medication side effects, hypersensitivity to external stimuli, resistance to dental care, and hyposensitivity to dental pain [248].

As noted above, oral bacteria (e.g., *H. parainfluenza*) or their metabolites can trigger inflammation and impact neuroimmune activity by crossing the BBB [249],[250]. Research reveals that BBB permeability is heightened in children with ASD [251], permitting oral bacteria to influence the CNS by disrupting metabolic activity or inducing inflammation [252]. Dysbiosis of OM can affect complex behaviors such as emotional behavior and anxiety in two ways [253]. First, transcriptional changes result in impaired microglial function, leading to an increase in BBB permeability for microbial metabolites. When periodontal inflammation occurs in children with ASD, it may cause leakage of LPS across the BBB, which can trigger an inflammatory response and disrupt metabolic activity in the CNS. Prolonged alteration of energy metabolism in oligodendrocytes, neurons, and microglia can cause structural modifications in the amygdala, cerebellum, hippocampus, and cortex, resulting in behavioral disturbances in children with ASD. The increase in LPS in children with ASD correlates with an increase in IL-6, which acts as both a pro-inflammatory cytokine and an anti-inflammatory myokine. In addition, inflammation in the developing brain causes synaptic dysfunction, resulting in vasopressin secretion that can also affect social behavior [250],[254]. The second pathway is through the gut–brain axis, which involves a link between the CNS and gut microbiome through direct neural activation. Prolonged OM dysbiosis can lead to gut microbiota dysbiosis, which affects various behavioral aspects, such as those related to emotions and anxiety-associated states [250],[255],[256].

The OM is altered in children with ASD. α-diversity, including indices of richness, diversity, and evenness, was significantly lower in individuals with ASD in comparison to healthy controls in dental samples. Nevertheless, none of these indices exhibited substantial differences in saliva samples, although a lower level of bacterial richness was observed [257]. The most prevalent phyla in both groups were Bacillota, Pseudomonadota, Actinomycetota, Bacteroidota, and Fusobacteriota, which accounted for more than 98% and 95% of the salivary and dental microbiota, respectively. At the same time, certain genera showed notable differences in their abundance between children with ASD and healthy controls. In particular, ASD was associated with increased abundance rates of *Streptococcus* and *Rothia* (in plaques) and *Haemophilus* (in saliva), and decreased abundance of *Prevotella* and *Selenomonas* (in plaques), *Actinomyces* and *Porphyromonas* (in saliva), and *Fusobacterium* (in plaques and saliva).

In a cross-sectional study conducted by Kong et al. [258], different characteristics of intestinal and salivary microbiota were detected between subjects with ASD and neurotypical controls. The α- and β-diversity of OM showed no significant variations between autistic and neurotypical individuals. At the genus level, the ASD and control groups shared 9 of the 10 most prevalent genera, such as *Haemophilus*, *Prevotella*, *Rothia*, *Fusobacterium*, *Streptococcus*, *Veillonella*, *Neisseria*, and a non-recognized genus within the *Neisseriaceae* family. Another study compared the OM of the tongues of children with ASD to healthy controls [259]. Regardless of grouping, *P. melaninogenica, H*. *parainfluenzae*, *R*. *mucilaginosa*, and *N. flavescens/subflava* were the most abundant species. Thirteen species and three genera showed differential abundance between the two groups. For instance, increased abundance of *Actinomyces* species (*A*. *odontolyticus* and *A. lingnae*) and decreased abundance of *Streptococcus vestibularis* and *Campylobacter concisus* was found in the group with ASD.

More recently, Manghi et al. [260] conducted a cross-sectional study in which OM composition could effectively differentiate individuals with ASD from neurotypical siblings. The relative abundance of certain microbial species showed strong correlations with developmental coordination and social communication issues, cognitive impairment, and restrictive behaviors such as those related to eating. With respect to OM, α-diversity did not show significant differences in ASD subjects compared to controls. In addition, 48.2% of the detected species were more prevalent in the saliva of children with ASD. In this regard, the five species more strongly associated with ASD were *Actinomyces johnsonii*, *Actinomyces hongkongensis, Cutibacterium acnes*, *Rothia dentocariosa*, and *Eikenella NML 130454*. In contrast, the remaining percentage was associated with neurotypicality, including *Prevotella pallens*, *Eubacterium sulci*, *Prevotella jejunii*, *Prevotella shaii*, and *Oribacterium parvum*. There are also substantial distinctions in metabolic pathways linked to serotonin, γ-aminobutyric acid (GABA), and dopamine degradation, which appeared to be mainly associated with cognitive deficits [261]. Additionally, a study reported differences related to diagnosis in OM composition between 80 autistic children and 40 age-matched typically developing peers [262]. The results indicated that the bacterial genera *Stomatobaculum*, *Solobacterium*, *Ruminococcaceae UCG.014*, *Campylobacter*, and *Tannerella* were notably more prevalent in autistic children than in children without autism. Moreover, the first three genera, which were substantially more prevalent in the ASD group, exhibited significant correlations with social difficulties reported by parents, repetitive and restrictive behaviors, and anxiety-associated behaviors reported by parents. Furthermore, these authors found associations of these five bacterial genera with clinical features. The oral abundance of all the genera was significantly linked to more social difficulties, while *Solobacterium* and *Ruminococcaceae UCG.014* were associated with more repetitive and restrictive behaviors, and *Solobacterium*, *Ruminococcaceae UCG.014*, and *Tannerella* were more linked to anxiety-like behaviors.

#### 6.2.2. Anxiety and depression

Anxiety disorders are defined by a range of symptoms such as excessive worry, fear in social and performance situations, panic attacks, and avoidant behaviors [263]. Within this context, although the exact pathogenesis of panic disorder is not fully understood, it is believed to be influenced by several factors, including genetic susceptibility, environmental exposures, neurotransmitter imbalances, and dysfunction of the amygdala [264].

Glutamate systems within cortico-limbic circuits engage with dopaminergic, GABAergic, serotoninergic, and other systems implicated in the stress response, suggesting a potential role in the onset of anxiety disorders [265]. Unlike glutamate, GABA serves as the main inhibitory neurotransmitter within the nervous system. Anxiety seems to result from a disruption between excitatory and inhibitory systems, leading to dysregulation [266].

The connection between panic disorder and OM has been reported in various studies [267]–[269]. Xie et al. [270] showed that the OM of individuals with panic disorder had higher evenness and higher abundance of the genera *Veillonella* and *Prevotella* and stated that OM could induce panic disorder via metabolic (arginine and proline) metabolism and inflammatory pathways. In this sense, panic disorder activates the HPA axis to activate cortisol secretion, which also affects the composition and metabolism of OM [271],[272]. In addition, great levels of cortisol released into the oral cavity, through the salivary glands, may affect oral health and OM within the context of anxiety disorders [273]. In a large-scale, population-based cohort of subjects, Malan-Müller et al. [268] found that anxiety disorder diagnosis was linked to a lower relative abundance of *Neisseria elongata* and to a greater relative abundance of *Oribacterium asaccharolyticum*.

Depression is a prevalent condition that significantly impairs psychosocial functioning and reduces quality of life [274]. It is primarily characterized by a persistent low mood but is often accompanied by distressing emotions, irritability, anxiety episodes, reduced performance in cognitive domains, apathy, social withdrawal, lack of motivation, anhedonia, hopelessness, psychomotor retardation, muscle hypotonia, and pervasive negative thoughts, including delusional beliefs in severe cases [275]. Neurotransmitters play a key role in mood disorders, with diminished dopamine signaling contributing to depressive symptoms [276]. Similarly, serotonin is implicated in mood disorders such as anxiety and depression. In this respect, although the serotonin hypothesis of depression has faced scrutiny, its influence on mood regulation is widely acknowledged [277]. The serotonin system may also serve as a pathway for circadian regulation of depression vulnerability [278]. In addition, depression is a major risk factor for suicide and involves complex pathogenic mechanisms, including hyperactivation of the HPA axis, inflammatory cytokines, and dysregulation of endogenous metabolites [279].

Members of the family *Spirochaetaceae* were positively linked to both anxiety and depression symptoms in adolescents [273]. A large number of other families were also positively associated with depression, but not with anxiety, such as *Veillonellaceae*, *Lachnospiraceae* XIV, Bacteroidales (F2), and *Actinomycetaceae*. At the genus level, *Lachnospiraceae G8*, *Bacteroidales bacterium GP2*, and *Mitsuokella* were positively related to depressive symptoms. In turn, at the species level, the genera positively linked to depression were *Prevotella salivae*, *Actinomyces* spp., *Prevotella* spp., *Lachnoanaerobaculum orale*, *Treponema* spp., *Mogibacterium* spp., *Mobiluncus mulieris*, *Selenomonas* spp., and *Fusobacterium periodonticum*. A total of 16 taxa significantly correlated with cortisol at the species level, including species linked to both anxiety and depression such as *Lachnoanaerobaculum orale*, *Selenomonas* spp., and *Streptococcus* spp. At the genus level, cortisol influenced the relationship between anxiety and the abundance of *Actinomyces* and *Rothia*, while at the species level, it affected associations with *R. mucilaginosa* and *Prevotella oulorum*. CRP moderated numerous links, such as those between depression symptoms and the prevalence of *Alloprevotella rava*, *Haemophilus* spp., *Neisseria* spp., and *Streptococcus* spp. Elevated cortisol levels have also been identified in the gingival crevicular fluid of women experiencing depression compared to healthy controls, correlating with increased inflammation and dental plaque formation [280]. Moreover, cortisol has been shown to modify microbial gene transcription, resulting in expression patterns similar to those found in periodontitis [281].

Wingfield et al. [282] investigated the composition of the salivary microbiota in a sample of depressed young adults. Notable variations in α- and β-diversity of the salivary microbiota were noted between depressed and healthy control cohorts. In addition, 21 bacterial taxa were identified to be distinctly prevalent in the cohort of depressed subjects, including increased abundance of *Prevotella nigrescens* and *Neisseria* spp., whereas 19 taxa presented a reduced abundance, including members of the genera *Rothia*, *Prevotella*, *Treponema*, *Haemophilus*, *Schaalia*, *Solobacterium*, *Neisseria*, *Lepotrichia*, *Veillonella*, and *Fusobacterium*. In a different study that employed a sample of 105 depressed cigarette smokers, Actinomycetota, Bacteroidota, Bacillota, Fusobacteriota, and Pseudomonadota were the most prevalent phyla, while *Veillonella*, *Streptococcus*, and *Haemophilus* were the most abundant genera in terms of oral microbiota [283]. These authors also reported that the group exhibiting higher depression possessed a greater OM diversity and richness. Using a genome-wide association study (GWAS), Li et al. [284] observed significant interactions between salivary-tongue dorsum microbiota in relation to anxiety and depression features. Importantly, a typical interaction was noted to be linked to anxiety and depression scores, specifically *Centipeda periodontii SGB 224* × *Granulicatella uSGB 3289*. Furthermore, these authors identified *Eggerthia*, a salivary microbiota, to be related to anxiety and depression. Interestingly, a higher relative abundance of *P. histicola* was evident in individuals with higher CESD depressive scores and those with poor psychological quality of life scores. Subjects with higher CESD scores also presented a greater relative abundance of *O. asaccharolyticum* and *Lancefieldella* spp. [268].

#### 6.2.3. Psychological stress

Stress is caused by a difficult situation or challenging event, known as a stressor, to which the body develops either a physiological or psychological reaction called the stress response [285]. Psychological stress arises when environmental demands overwhelm an individual's ability to adapt effectively [286]. Over time, chronic stress may disrupt behavioral, physiological, and emotional processes, influencing both the onset and progression of diverse diseases [287]. Modern lifestyles, marked by elevated stress levels, intake of processed foods, and overuse of antibiotics, along with environmental fluctuations, such as heightened pollution, urbanization, and climate change, have driven the human microbiota toward an industrialized composition [288].

The experience of stress varies greatly from person to person, and when a stressor is evaluated, stress axes are activated, implicating brain regions such as the amygdala, hippocampus, and prefrontal cortex. The two primary stress-response systems are the sympathetic nervous system, also known as the sympatho-adrenomedullary (SAM) axis, which releases catecholamines, and the HPA axis, which triggers the release of cortisol. After exposure to stress, activation of the HPA axis mediates the “fight or flight” response, promoting cortisol and glucose secretion. The complexity of the stress response is not only limited to neuroanatomy or to the mediators of the SAM and HPA axes but also varies according to the duration of stressor exposure and to the timing, as well as its immediate and long-term repercussions [289]. Acute stress-induced inflammation is regulated by catecholamines, which protect the organism from potential damage or infection. Conversely, chronic stress triggers the HPA axis to release cortisol to mitigate excessive inflammation. This process includes pro-inflammatory cytokines that form a self-regulating feedback mechanism at different levels of the HPA axis [290]. The regulation of catecholamines seems to act as a protective mechanism, rapidly mobilizing immune responses to mitigate potential harm [285],[291]. Nevertheless, for preventing damage caused by an excessive inflammatory response, the stress response also activates the HPA axis, stimulating the cortisol release [292]. This activation primarily occurs via the secretion of pro-inflammatory cytokines such as TNF-α, IL-1, and IL-6. These cytokines create a self-regulating feedback loop by affecting different levels of the HPA axis, including the pituitary and adrenal glands as well as the paraventricular nucleus. As a consequence of intermittent or sustained stress, the HPA axis can become dysregulated, characterized by persistently elevated cortisol levels that may last for years, a condition called hypercortisolism, which is indicative of an overreactive HPA axis or hypercortisolemia. Hypercortisolism can result in the depletion of spleen, thymic, and lymphoid tissues, impaired cytokine production, and a widespread suppression of cellular immunity. Stress-induced hypercortisolism is counteracted by compensatory inhibition of cortisol's immunoregulatory effects, leading to a reduction in the levels of cortisol [293]. Additionally, the influence of cortisol on target tissues may be modulated by cortisol-induced downregulation of the HPA axis and by glucocorticoid receptor (GR) activity. Moreover, hypocortisolism and GR resistance are reciprocally associated with increased inflammatory activity and subsequent tissue damage [294].

Diverse studies have proposed links between oral health, the immune system, and several psychosocial factors [295]. Specifically, psychosocial stress has been associated with poorer oral health, although the exact psychophysiological mechanisms underlying this connection remain broadly unclear [296]. The direct immuno-neuroendocrine influence was underlined by changes in several salivary biomarkers (e.g., TNF-α, IL-6, IL-1β, α-amylase) of healthy individuals after specific psychological conditions. This implies a direct biochemical impact of psychosocial factors on the oral ecosystem [297]. The psychosocial components of periodontitis have been extensively studied, with stress identified as a contributor to its initiation and development. Acute stress may impair cellular immunity, whereas prolonged stress results in systemic immune dysregulation. Salivary concentrations of β-endorphin and cortisol, along with psychological states like anxiety and depression, are linked to periodontal health outcomes [298].

A considerable amount of research has focused on the in vitro effects of stress-associated proteins and hormones, including adrenaline, noradrenaline, mucin, and cortisol, on OM alteration. For example, Roberts et al. [299] found that adrenaline and noradrenaline stimulated the growth of *Campylobacter*, *Eikenella*, and *Actinomyces* and suppressed the growth of *Bacteroides forsythus* and *P. gingivalis* isolated from the subgingival microbial complex. In addition, cortisol, adrenaline, and noradrenaline substantially diminished the growth of *F. nucleatum* after 12 hours of treatment [300]. In contrast, the growth of the periodontal pathogen *Porphyromonas endodontalis* was suppressed after treatment with cortisol, adrenaline, and noradrenaline for 24 hours, although no inhibitory effects were noted in the case of *P. intermedia* and *P. gingivalis* [300]. Changes in the gene expression profiles of the microbiome, resembling those seen in periodontitis, were detected through a metatranscriptomic analysis of samples obtained from dental plaque exposed to cortisol [281]. These authors reported that *Leptotrichia goodfellowii*, belonging to the phylum Fusobacteriota, presented a substantially greater abundance of transcripts in the plaque samples treated with cortisol. However, the response of other members of this phylum, such as *F. nucleatum*, to stress-related proteins and hormones is very different. Although mucin, a salivary protein linked to stress, has been shown to enhance the viability of *F. nucleatum*, cortisol did not seem to influence this bacterium [301].

The role that psychological stress plays in OM has been investigated in several clinical studies. For example, Nani et al. [302] examined the connections between salivary bacteria, oral emissions of volatile sulfur compounds, and academic-associated persistent stress. The main findings of these authors were that the academic-associated persistent stress enhanced salivary *Solobacterium moorei* levels. *S. moorei* showed a correlation with *F. nucleatum*, which in turn was linked to hydrogen sulfide levels in the stress group, contributing to halitosis. Differential salivary microbiota diversity in relation to the affective state was found by Kohn et al. [303]. The high-stress group exhibited greater α-diversity compared to the low-stress group. The taxa most strongly linked to host distress encompassed *Haemophilus*, *Selenomonas*, and *Leptotrichia*, whereas the taxa with the weakest association were predominantly from the genus *Prevotella*. Israeli veterans diagnosed with post-traumatic stress disorder (PTSD) following participation in the Lebanon War exhibited OM dysbiosis, and decreased bacterial species (*Alloprevotella* spp., *Caprocytophaga* spp., and *Selenomonas noxia*) were correlated with PTSD severity, including intrusiveness, arousal, and reactivity, as well as with other psychopathological symptoms such as memory impairments, idiopathic pain, hostility, and anxiety. Similarly, in a study on Israeli veterans of the 1982 Lebanon War with PTSD symptoms, Levert-Levitt et al. [304] reported that a specific microbiota signature (i.e., decreased levels of the bacteria sp_HMT_914, 332, and 871, as well as *S. noxia*, alongside an increase in members of the phylum Bacteroidota) correlated with PTSD severity. This correlation was observed in relation to symptoms of arousal, intrusiveness, and reactivity, as well as additional psychopathological manifestations, including anxiety, hostility, idiopathic pain, and memory impairment. An overall increase in bacterial species richness was found at the end of a training week for military medical students [305], and several different bacterial taxa were identified from the beginning to the end of data collection, including the genera *Corynebacterium, Eikenella, Tannerella, Rothia, Capnocytophaga*, *Leptotrichia, Haemophilus, Stomatobaculum, Aggregatibacter*, and *Porphyromonas*, and also members of the families *Actinomycetaceae* and *Pasteurellaceae*.

More recently, groups of pregnant women with high and low PTSD symptoms differed in β-diversity, indicating variations in OM composition [306]. Discriminant analysis revealed differentially abundant microbiota between women experiencing high stress and low life stress, as well as those with PTSD. Specifically, members of the phylum Pseudomonadota were more prevalent in women with high recent life stress, and the genus *Eikenella* was increased with high PTSD. Finally, Charalambous et al. [307] examined the interplay between OM and the stress response in a cohort of adults who either experienced institutionalization and adoption or were non-adopted controls. The authors identified the following 12 bacterial taxa: Absconditabacteriales, *Acinetobacter*, *Clostridia UCG14*, *Campylobacter, Cardiobacterium, Oxalobacteraceae, Sphingomonas, Bradyrhizobium, Comamonadaceae, Flavobacterium, Methylobrubrum*, and *Paucibacter*, which interacted with the host cortisol and glucose response to stress and strongly influenced the clearance and intensity of cortisol and glucose after exposure to stress. Evidence indicates that exposure to high levels of cortisol leads to upregulation of virulence factors (LPS, fimbriae, or gingipains) in the periodontitis-related bacterial taxa *Fusobacterium* and *Poprhymonas*, leading to a global change in the composition toward a pathogenic type [198],[308],[309]. This capacity of OM to adapt to the hormonal response of the host is partially attributed to resilience mechanisms that determine the community composition and drive homeostasis [6]. During HPA activation and stress response, catecholamines are released in addition to cortisol. Several studies have reported that these stress hormones can foster or suppress the growth of oral microbiota associated with periodontitis; while it is clear that stress induces taxonomic shifts in the composition of OM [6],[310], the directionality of the interaction remains unclear. The OM has been shown to be implicated in the modulation of neurological processes, shaping cognition and behavior via interaction with the neuroendocrine system [198]. This modulation could partly result from metabolites or other small molecules released by OM and absorbed by the host [311].

#### 6.2.4. Bipolar disorder

Bipolar disorder (BD) is a psychiatric condition marked by significant mood fluctuations, including manic or hypomanic episodes and also periods of depression [312]. Patients with BD typically have deficient oral hygiene, which may be linked to low self-care, alcohol consumption, smoking, and the side effects of psychotropic drugs. Potential mechanisms that explain this connection include the influence of inflammatory mediators, modulation of the neurotransmitter system, direct impact of OM, and interactions with the vagus nerve and with the HPA axis [313]. Research on the relationship between BD and OM remains limited. Cunha et al. [314] found that BD is related to an increased risk of periodontitis, a greater prevalence of microbial pathogens, and an elevated bacterial load, including species such as *A. actinomycetemcomitans* and *P. gingivalis*. The increased prevalence of periodontitis and the bacterial load for *A. actinomycetemcomitans* and *P. gingivalis* were particularly pronounced during depressive phases, as compared to manic or euthymic phases [314].

#### 6.2.5. Schizophrenia

Schizophrenia is a chronic mental disorder encompassing three symptom categories: (i) positive symptoms such as delusions, hallucinations, and disorganized thinking; (ii) negative symptoms such as emotional flattening, diminished affect, and social withdrawal; and (iii) cognitive symptoms such as difficulties with learning and attention [315]. Research has shown that periodontal disease causes neuroinflammation by releasing inflammatory cytokines within the CNS and by stimulating immune and microglial cells, potentially being implicated in the mechanism by which inflammation influences schizophrenia [316]. Compared to the controls, patients with schizophrenia have a distinct microbial composition based on α- and β-diversity, and these differences are linked to symptom severity [317]. Interestingly, it has been noted that oral metabolism disorders arise prior to the development of schizophrenia and are implicated in its pathogenesis via the peripheral inflammatory response and redox system [318]. An imbalance in the OM in saliva may also lead to the pathogenesis of schizophrenia by inducing oxidative stress, dysregulated tyrosine metabolism, peripheral inflammation, and BBB permeability [318]. Regarding the composition of OM, several review studies have been conducted. Castro-Nallar et al. [319] found differences between both groups (PWS and HC) at phylum and genus levels, with the fungi phylum Ascomycota being more abundant in PWS than HC. Lactic acid bacteria were relatively more abundant in PWS, including species of lactobacilli, bifidobacteria, and *Eubacterium halii*, which have been shown to modulate chronic inflammation. Dickerson et al. [320], in a review study, reported that among 25 differentially abundant microbial species (bacteria and fungi), 6 were significantly more prevalent in schizophrenia cases compared to controls after adjusting for relevant covariates. Lactic acid bacteria, such as *Lactobacillus* and *Bifidobacterium*, were notably enriched in schizophrenia, with *Lactobacillus gasseri* being 400 times more abundant in patients than in controls. Yolken et al. [321] found an altered β-diversity in PWS and mania as compared to HC and reported that the oropharyngeal microbiota of PWS and subjects with mania differed from HC. Three of the taxa, *Prevotella*, *Weeksellaceae*, and *N. subflava*, were reduced in PWS or mania in comparison to that of controls, while streptococci were increased in these groups. Furthermore, Qing et al. [322] found that the salivary microbiome of PWS was marked by greater α-diversity and lower β-diversity heterogeneity than that of controls. In addition, Pseudomonadota, the predominant phylum, was depleted, while Bacillota and the Bacillota/Pseudomonadota ratio were enriched from controls to schizophrenic patients. H_2_S-producing bacteria showed enrichment specific to disease stages and may serve as potential diagnostic biomarkers for schizophrenia. More recently, several studies have suggested that components of the OM could serve as potential diagnostic biomarkers for schizophrenia. For example, Ling et al. [323] reported that schizophrenia-associated oral dysbiosis, characterized by increased levels of *Streptococcus* and *Fusobacterium*, and decreased levels of *Prevotella* and *Veillonella*, may act as a potential biomarker for schizophrenia. Furthermore, an elevated *Streptococcus*/*Prevotella* ratio could indicate oral dysbiosis, with associations to immunological profiles, as well as lipid and amino acid metabolism. Regarding oral fungal communities, Liu et al. [324] identified two distinct mycotypes in schizophrenia patients: one showing an increase in the genus *Malassezia* and the other showing reduced *Candida* levels. Moreover, schizophrenia patients exhibited signs of immunological dysfunction, characterized by elevated levels of pro-inflammatory cytokines such as IL-6 and TNF-α, and chemokines such as MIP-1α and MCP-1. Notably, the *Malassezia* mycotype was positively correlated with peripheral pro-inflammatory cytokines, while the *Candida* mycotype showed a negative correlation with these cytokines.

### 6.3. Other brain-related conditions

#### 6.3.1. Down's syndrome

Down's syndrome (DS) is a genetic disorder characterized by mild to moderate intellectual disability and distinct facial features. DS is also associated with specific oral characteristics, including a decreased prevalence of dental caries but an increased prevalence of periodontitis and gingivitis when compared to controls. Nevertheless, the overall composition of OM in individuals with DS, as well as how it varies with various factors such as host age or oral pH, remain insufficiently explored [325]. Although several studies have shown that the DS population develops earlier and more extensive periodontal destruction, Reuland-Bosma et al. [326] reported an absence of specific microbiota in adults with DS compared to controls. Despite this result, Amano et al. [327] identified *P. gingivalis* involved in gingival inflammation and plaque accumulation in individuals with DS; also, salivary *S. mutans* was higher in children and adolescents with DS and mental retardation than in controls [328]. Khocht et al. [329] reported that the prevalence of periodontal bacterial pathogens in individuals with DS may vary depending on age. The subgingival microbiological profiles of adults with DS and periodontitis resemble those found in individuals with chronic periodontitis. Specifically, *Prevotella nigrescens*, *Peptostreptococcus micros*, and *E. corrodens* are substantially more prevalent in young adults with DS, while *P. gingivalis*, *A. actinomycetemcomitans*, *C. rectus*, *P. intermedia*, and *C. sputigena* are more common during adolescence. In addition, *Actinomyces naeslundi* and *T. forsythia* are consistently found in individuals with DS, regardless of age. These authors also noted notable distinctions between the groups for certain bacterial species, including *T. forsythia, P. intermedia*, *A. actinomycetemcomitans*, *P. gingivalis, S. noxia, C. acnes, S. oralis*, *S. gordonii*, *S. mitis*, *Treponema socranskii*, and *S. constellatus*. However, the majority of detected bacteria are opportunistic, indicating that factors such as hand contamination (e.g., finger biting or thumb sucking) may play a role. More recently, Willis et al. [330] reported that DS is linked to lower salivary pH and a reduced OM diversity, characterized by lower levels of *Atopobium*, *Alloprevotella*, and *Candidatus Saccharimonas aalborgensis*, as well as by higher levels of *Gemella*, *Kingella, Staphylococcus, Cardiobacterium, Actinobacillus*, and *Rothia*, along with a greater prevalence of *Candida parapsilosis and Candida dubliniensis*. However, other authors have reported that the taxonomic analysis of OM showed notable differences in the relative abundance levels of specific bacteria between the DS and non-DS groups. Cuenca et al. [331] studied various periodontal conditions, including 62 cases of DS-periodontal health, 34 cases of DS-gingivitis, and 28 cases of DS-periodontitis. *T. forsythia* was the most prevalent species in all groups, with significantly higher percentages in DS-periodontitis compared to DS-gingivitis and DS-periodontal health. Furthermore, the frequency detection, counts, and proportions of *P. gingivalis*, *P. intermedia*, *T. forsythia*, and *E. corrodens* showed a progressive increase that correlated with the deterioration of periodontal status. In addition, Mitsuhata et al. [332] found that children with DS do not exhibit red complex microorganisms (*P. gingivalis*, *T. forsythia*, and *T. denticola*) but have a unique OM that may influence the development of dental diseases common in children with the syndrome, characterized by the presence of *Corynebacterium*, *Abiotrophia*, and *Lautropia*. However, other studies indicate that the oral bacterial composition in individuals with DS may resemble that of non-syndromic individuals with periodontitis [333]. Conversely, once periodontitis is present, the subgingival microbiota of subjects with DS shows a greater abundance of *Porphyromonas*, *Treponema*, *Aggregatibacter*, and *Tannerella*, along with a lower abundance of genera such as *Filifactor*, *Fretibacterium*, and *Desulfobulbus*, suggesting a microbial profile analogous to that found in non-syndromic individuals affected by periodontitis [334]. Previously, it was noted that the prevalence of periodontal bacterial pathogens may vary with the age of individuals with DS. *Peptostreptococcus micros*, *P. nigrescens*, and *E. corrodens* are significantly more prevalent in young adults with DS, while *C. rectus*, *A. actinomycetemcomitans*, *P. gingivalis*, *P. intermedia*, and *C. sputigena* are more commonly found during adolescence. Furthermore, *T. forsythia* and *A. naeslundi* are consistently observed in subjects with DS across all age groups.

#### 6.3.2. Cerebral palsy and epilepsy

Children with cerebral palsy and epilepsy are also affected by periodontitis and dental caries. In fact, clinical research indicates that up to 86% of children diagnosed with cerebral palsy have mild-to-moderate gingivitis [335], and a similar percentage have dental caries [336]. One study found decreased levels of Bacillota and Bacteroidota, alongside elevated levels of Actinomycetota, in the mouth of children with cerebral palsy and epilepsy compared to healthy children [337]. A comparative analysis of the supragingival plaque microbiota in children with cerebral palsy and severe caries versus those without cerebral palsy and severe caries showed a higher abundance of *P. intermedia*, *Fusobacterium nucleatum, C. rectus, P. endodontalis, Streptobacillus moniliformis*, *Catonella morbi, Parvimonas micra*, *Porphyromonas canoris*, and *Alloprevotella tannerae* in the former group. These bacteria may contribute to the pathogenesis of cerebral palsy via the oral–gut–brain axis [338]. Compared to individuals without epilepsy, epileptic subjects present heightened α-diversity in their OM, alongside significant β-diversity differences between the groups. In this regard, epileptic patients have a marked increase in the abundance of 26 genera, such as *Kluyvera*, *Granulicatella*, and *Streptococcus*, while experiencing a substantial reduction in 14 genera, including *Schaalia*, *Neisseria*, and *Peptostreptococcus* [339]. A recent Mendelian randomized analysis in East Asian children with epilepsy was conducted by Zhao et al. [340]. These authors found that 14 oral microbial taxa, such as *S. mitis*, *S. pneumoniae*, and *Haemophilus* spp., were positively associated with epilepsy, while members of the genera *Fusobacterium* and *Aggregatibacter* were negatively related to epilepsy.

## 7. Discussion

The World Health Organization considers oral health as a key indicator of overall health, well-being, and quality of life. Besides, it states that oral health shares modifiable risk factors with the leading non-communicable diseases, such as cardiovascular diseases, cancer, chronic respiratory diseases, diabetes, or autoimmune diseases, to name a few [341]. In recent decades, several studies have supported the significant role of the OM and the oral–gut–brain axis in the etiology and development of various psychological and psychiatric disorders [188],[190]. While the influence of OM on mental health requires further clarification, oral health is acknowledged as a pivotal factor in overall well-being and quality of life [342]. Oral diseases can profoundly impact the functional and psychosocial dimensions of individuals [343], leading to a diminished quality of life in those affected by common dental conditions [344],[345], as well as in patients with malignant diseases such as oral cancer [346].

The use of next-generation sequencing methods has led to important advances in the study of the microbiome in the human body [347]. From a clinical and human health perspective, OM, in addition to causing oral diseases, will become a new focus. In this regard, the importance of OM management has increased due to the growing aging population, improved medical treatment prognosis, and infection control in immunocompromised patients [348]. Nevertheless, there is still exiguous evidence on the identification of OM for disease prevention and health promotion. In this review, we have highlighted the involvement of specific oral pathogenic bacteria in systemic diseases [142] and mental health [189]. Thus, it is plausible to state that OM play a pivotal role in both oral and systemic health, including mental health, and that it is potentially influenced by diverse factors such as diet, age, genetics, drug consumption, and other environmental exposures, which shape its composition and diversity. Although primarily studied for its local effects, growing evidence suggests the significant impact of OM on brain health. Direct and indirect related pathways provide potential routes through which the OM can influence neuroinflammation, disrupt the BBB, and contribute to altered cognitive function. Regarding this, certain oral pathogens have been shown to invade the bloodstream and affect neuronal processes. Specifically, changes in OM may lead to gut dysbiosis, which in turn leads to low-grade inflammation in the gut and elevated permeability of biological barriers, including the BBB [195]. Neuroinflammation and cognitive impairment seem to be derived factors of OM dysbiosis based on the results revealed in various studies [196],[197]. Moreover, oral pathogens such as *P. gingivalis* lead to an “oralization” of the gut microbiota [349],[350], a mechanism that causes gut inflammation and may be related to the emergence and maintenance of neuroinflammation [194],[351] via the translocation of oral/intestinal toxic bacterial proteases to the brain [188],[352]. Therefore, substances with anti-inflammatory properties may help to ameliorate the development or alleviate the symptoms of certain mental disorders [353]. It has been stated that a high intake of docosahexaenoic acid (DHA) may provide neuroprotection during the early stages of the onset of dementia-related cognitive deficits [354]. In the same vein, several components of vegetarian diets and fermented vegetables, such as the fatty acids of long-chain ω-3 derivatives and plant polyphenols, including resveratrol, have been associated with beneficial effects on brain health [355]. Ribeiro-Vidal et al. [356] examined the in vitro antimicrobial properties of two ω-3 fatty acids, DHA and eicosapentaenoic acid (EPA), on a subgingival biofilm. The findings indicate that DHA and EPA significantly reduced the harmful bacterial strains contemplated, such as *F. nucleatum*, *P. gingivalis*, *A. actinomycetemcomitans*, and *V. parvula*. However, this study used an in vitro model, and randomized controlled trials are needed to ensure the beneficial properties of these substances on oral pathogens and mental health. In addition, plant polyphenols, such as anthocyanins, have shown beneficial effects in preventing and ameliorating certain clinical manifestations of progressive AD [357],[358]. In addition, polyphenols have been suggested as potential therapeutic agents in the treatment of oral infectious diseases [359],[360]. Another approach to control and reduce inflammation is the use of antimicrobial peptides, which can affect oral cavity homeostasis through the broad or selective killing of bacterial pathogens and through their immunomodulatory properties, which can impact both the innate and adaptive immune response [361].

Although several other therapeutic tools have been used for oral diseases, including probiotic, prebiotic, synbiotic, and postbiotic treatment [362], as well as photodynamic [363], quorum quenching [364], and phage therapy [365], to date these treatments have been hardly applied in mental health related to oral microorganisms. Maitre et al. [366] reported a limited development concerning the use of prebiotics and/or probiotics for modulating OM dysbiosis, which may be implicated in the onset and progression of mental disorders. Therefore, more efforts need to be made in the future to find an effective treatment for mental disorders associated with oral pathogens, as exemplified by the use of psychobiotics within the context of gut dysbiosis, which have been applied in the treatment of adverse mental states such as stress, anxiety, and depression through the modulation of the gut microbiome [367].

Furthermore, it is worth highlighting an important psychosocial aspect of the relationship between oral and mental health, which extends beyond physiological factors. Poor oral health and visible dental issues can contribute to social rejection in interpersonal interactions, with the level of visibility being a key factor in the intensity of discrimination. While dental problems are directly visible, bad breath, although not seen, can still provoke strong negative reactions in close proximity. This dynamic can be likened to the stigma associated with skin conditions, such as acne or psoriasis, which are also linked to microbiome dysbiosis and can contribute to a major risk of bullying victimization and social rejection [368]. In this respect, given that individuals with lower socioeconomic status often experience poorer oral health [75], this situation could exacerbate the risk and severity of social exclusion, creating a compounded disadvantage for them.

As with any study of this nature, the present narrative review is subject to various limitations, as follows: (i) The oral cavity contains multiple ecological niches, each characterized by distinct microbial communities, contributing to the considerable diversity of OM. This inherent complexity makes it challenging to draw definitive conclusions. (ii) It is important to note that the studies included in this review primarily focus on bacterial communities. However, emerging research on the oral mycobiome and viral communities indicates that these microorganisms may also play a pivotal role in the regulation of OM homeostasis. (iii) Several of the studies reviewed are cross-sectional or exploratory, with small sample sizes, thus complicating the establishment of causal relationships between the examined factors. (iv) A significant limitation across the studies is the lack of standardized protocols for sample collection, sequencing, and data analysis, which impedes meaningful comparisons of results across different investigations. (v) Finally, the considerable variability in sequencing technologies and molecular techniques employed complicates the interpretation and comparison of findings across studies.

## 8. Conclusions

A substantial body of evidence indicates that gut microbiota dysbiosis plays a role in the onset and progression of various mental disorders. In contrast, research on the microbiota of other host-associated sites, such as the oral cavity, remains relatively underexplored. Investigating OM is particularly important, given its potential to interact with the gut microbiota via multiple pathways within the intestinal tract, as well as its direct influence on brain functions via the trigeminal nerve and olfactory system. The complex balance of syntrophic and antagonistic interactions within OM, along with processes like horizontal gene transfer, underscores the need for a metabolomic approach to fully understand its diversity and functionality. This highlights the importance of considering a wide range of factors, particularly those related to diet and lifestyle, when examining the determinants that shape OM and its potential impact on overall health. Chronic oral diseases can impair the oral mucosal barrier, allowing microorganisms and endotoxins to enter the bloodstream, triggering systemic inflammation and affecting the BBB. This pathway can lead to neuroinflammation and cognitive dysfunction and contribute to the onset and development of mental health conditions. Additionally, OM may influence brain function through the translocation of oral bacteria to the gut, driving persistent inflammation and thereby affecting brain health. Multiple studies suggest a potential relationship between OM and various mental and neurodegenerative disorders, such as AD, PD, anxiety, and depression. However, further research is needed to strengthen the evidence surrounding these associations and to fully clarify the underlying mechanisms linking OM to these conditions. Given the promising implications, future research should focus on elucidating the biological mechanisms through which alterations in OM influence the development and progression of determinate neurodegenerative and neuropsychiatric disorders. Additionally, identifying reliable biomarkers linked to OM could enhance early detection, diagnosis, and monitoring of these conditions. There is considerable promise in microbiota-targeted interventions, such as plant-based diets, probiotics, prebiotics, synbiotics, microbiome-derived metabolites, and fecal microbiota transplantation, for the treatment and prevention of these disorders. Furthermore, longitudinal research tracking changes in OM and their associations with brain-related conditions will be pivotal for translating microbiota-based interventions into clinical applications, marking a significant advancement within this field.

## Use of AI tools declaration

The authors declare they have not used Artificial Intelligence (AI) tools in the creation of this article.
